# Tidal volume challenge–induced hemodynamic changes can predict fluid responsiveness during one-lung ventilation: an observational study

**DOI:** 10.3389/fmed.2023.1169912

**Published:** 2023-08-09

**Authors:** Yang Zhang, Yinyin Ding, Jiatong Zhang, Tianfeng Huang, Ju Gao

**Affiliations:** Department of Anesthesiology, Northern Jiangsu People's Hospital, Yangzhou, China

**Keywords:** tidal volume challenge, one-lung ventilation, fluid responsiveness, stroke volume, thoracic surgery

## Abstract

**Background:**

To evaluate the ability of tidal volume challenge (V_T_C)-induced hemodynamic changes to predict fluid responsiveness in patients during one-lung ventilation (OLV).

**Methods:**

80 patients scheduled for elective thoracoscopic surgery with OLV were enrolled. The inclusion criteria were: age ≥ 18 years, American Society of Anesthesiologists physical status I-III, normal right ventricular function, normal left ventricular systolic function (ejection fraction ≥55%), and normal or slightly impaired diastolic function. The study protocol was implemented 15 min after starting OLV. Simultaneous recordings were performed for hemodynamic variables of diameter of left ventricular outflow tract, velocity time integral (VTI) of aortic valve, and stroke volume (SV), and ΔSV-V_T_C, ΔVTI-V_T_C, and ΔMAP-V_T_C were calculated at four time points: with V_T_ 5 mL/kg (T1); after V_T_ increased from 5 mL/kg to 8 mL/kg and maintained at this level for 2 min (T2); after V_T_ was adjusted back to 5 mL/kg for 2 min (T3); and after volume expansion (250 mL of 0.9% saline infused over 10–15 min) (T4). Patients were considered as responders to fluid administration if SV increased by ≥10%. Receiver operating characteristic (ROC) curves for percent decrease in SV, VTI, and MAP by V_T_C were generated to evaluate their ability to discriminate fluid responders from nonresponders.

**Results:**

Of the 58 patients analyzed, there were 32 responders (55%) and 26 nonresponders (45%). The basic characteristics were comparable between the two groups (*p* > 0.05). The area under the curve (AUC) for ΔSV-V_T_C, ΔVTI-V_T_C, and ΔMAP-V_T_C to discriminate responders from nonresponders were 0.81 (95% CI: 0.68–0.90), 0.79 (95% CI: 0.66–0.89), and 0.56 (95% CI: 0.42–0.69). The best threshold for ΔSV-V_T_C was −16.1% (sensitivity, 78.1%; specificity, 84.6%); the best threshold for ΔVTI-V_T_C was −14.5% (sensitivity, 78.1%; specificity, 80.8%).

**Conclusion:**

Tidal volume challenge–induced relative change of stroke volume and velocity time integral can predict fluid responsiveness in patients during one-lung ventilation.

**Clinical Trial Registration:** Chinese Clinical Trial Registry, No: chictr210051310.

## Introduction

Perioperative hemodynamic optimization is essential to prevent tissue underperfusion and fluid overload in patients with acute circulatory failure in the intensive care unit and operating room (1). Numerous studies have shown that, in critically ill and surgical patients, unnecessary perfusion increases morbidity, mortality, and length of hospital stay (2–10). Only in cardiac preload–dependent conditions, i.e., the rising part of the Frank–Stalin curve, does stroke volume (SV) increase after volume expansion. However, this occurs in only about 50% of intensive care and surgical patients (11, 12). In preload-independent conditions, i.e., the plateau branch of the Frank–Staling curve, volume expansion does not increase SV and may even increase the risk of adverse effects.

A proper fluid management strategy is crucial for patients undergoing thoracic surgery. Oxidative stress during one-lung ventilation (OLV) and after pulmonary resuscitation may cause pulmonary edema (13, 14). Pulmonary surgery can also lead to local edema via release of proinflammatory cytokines. In 2019, the Enhanced Recovery after Surgery Society and the European Society of Thoracic Surgeons recommended restrictive fluid therapy during lobectomy (15). However, there is concern that excessive restriction of fluid infusion may lead to hypovolemia and impaired tissue perfusion, organ dysfunction, and acute kidney injury. Therefore, assessment of fluid responsiveness is extremely important for hemodynamic management during OLV. As is well known, volume responsiveness assessment is the cornerstone of fluid therapy.

Traditional static indicators such as central venous pressure and pulmonary artery wedge pressure do not accurately reflect volume responsiveness (16, 17). Dynamic indicators such as stroke volume variation (SVV) and pulse pressure variation (PPV) are currently recognized as the best objective indicators of volume responsiveness of patients on conventional mechanical ventilation (16, 18). But the pathophysiological changes caused by opening the nonventilated side of the chest cavity during thoracic surgery, OLV, and lung-protective ventilation strategies with small tidal volumes can limit the usefulness of these dynamic indicators (19, 20). Changes in intrathoracic pressure affect the amount of blood returned to the heart, which in turn leads to changes in hemodynamics. Some authors have applied functional hemodynamic tests in the attempt to overcome the above limitations (21–23). One of these tests—the tidal volume challenge (V_T_C) test—which assesses changes in hemodynamics following an increase in the tidal volume from 6 mL/kg to 8 mL/kg for 1 min, has been shown to reliably predict fluid responsiveness in patients undergoing neurosurgery (24). To our knowledge, no studies have evaluated the usefulness of V_T_C for predicting fluid responsiveness during OLV. We hypothesized that the hemodynamic changes induced by temporarily increasing tidal volume from 5 mL/kg to 8 mL/kg could predict fluid responsiveness in patients receiving OLV. This study was designed to determine whether tidal volume challenge (V_T_C)-induced hemodynamic changes can predict fluid responsiveness in patients during OLV.

## Methods

### Study design

This study was approved by the hospital ethics committee (approval No: 2021ky244) and registered in the China Clinical Trial Registration Center (Registration No: chictr210051310). Written informed consent was obtained from all patients.

For this prospective trial, we enrolled 80 patients scheduled for elective thoracoscopic OLV surgery (thoracoscopic lobectomy, segmental lung resection, lung wedge resection, mediastinal tumor resection, chest wall lesion resection). The inclusion criteria were: 1) age ≥ 18 years; 2) American Society of Anesthesiologists (ASA) physical status category I-III; 3) normal right ventricular function; 4) normal left ventricular systolic function (ejection fraction ≥55%); and 5) normal or only slightly impaired diastolic function. The exclusion criteria were: 1) preoperative ejection fraction <55%; 2) body mass index >30 kg·m^−2^; 3) arrhythmia; 4) use of vasoconstrictor or inotropic drugs before or during V_T_C; 5) intracranial hypertension (clinical symptoms and signs, or imaging manifestations); 6) asthma or preoperative pulmonary dysfunction (forced expiratory volume in 1 s < 50% of predicted value); or 7) contraindications for transesophageal ultrasound (e.g., esophageal space occupying lesions, recent postoperative esophagectomy).

### Perioperative management

All patients received standard intraoperative monitoring (heart rate, peripheral oxygen saturation [SpO_2_], continuous electrocardiographic, and noninvasive blood pressure monitoring). General anesthesia was induced, after preoxygenation, with propofol, midazolam, sufentanil, and atracurium *cis*-benzene sulfonate, and maintained with sevoflurane, remifentanil, dexmedetomidine, and atracurium *cis*-benzene sulfonate. The depth of anesthesia was controlled to maintain a bispectral index of 45–60 throughout the duration of surgery.

After the airway was secured, the patient was turned to the lateral position, and OLV was initiated with lung-protective ventilation (tidal volume, 5 mL/kg of ideal body weight [IBW]; positive end-expiratory pressure [PEEP], 5 cm H_2_O). Ventilatory rate was controlled to keep end-tidal CO_2_ within 35–40 mm Hg, and peak inspiratory pressure below 30 cm H_2_O. Inspired oxygen concentration was 100% at the start of OLV and gradually decreased, ensuring that SpO_2_ remained >95% throughout OLV. After anesthesia induction, a 20G cannula was inserted into the radial artery for invasive blood pressure monitoring, and an esophageal ultrasonic Doppler probe was placed through the mouth. Throughout the study duration, Ringer solution was administered intravenously at a rate of 3 mL·kg^−1^·h^−1^.

### Study protocol

**Figure 1:**
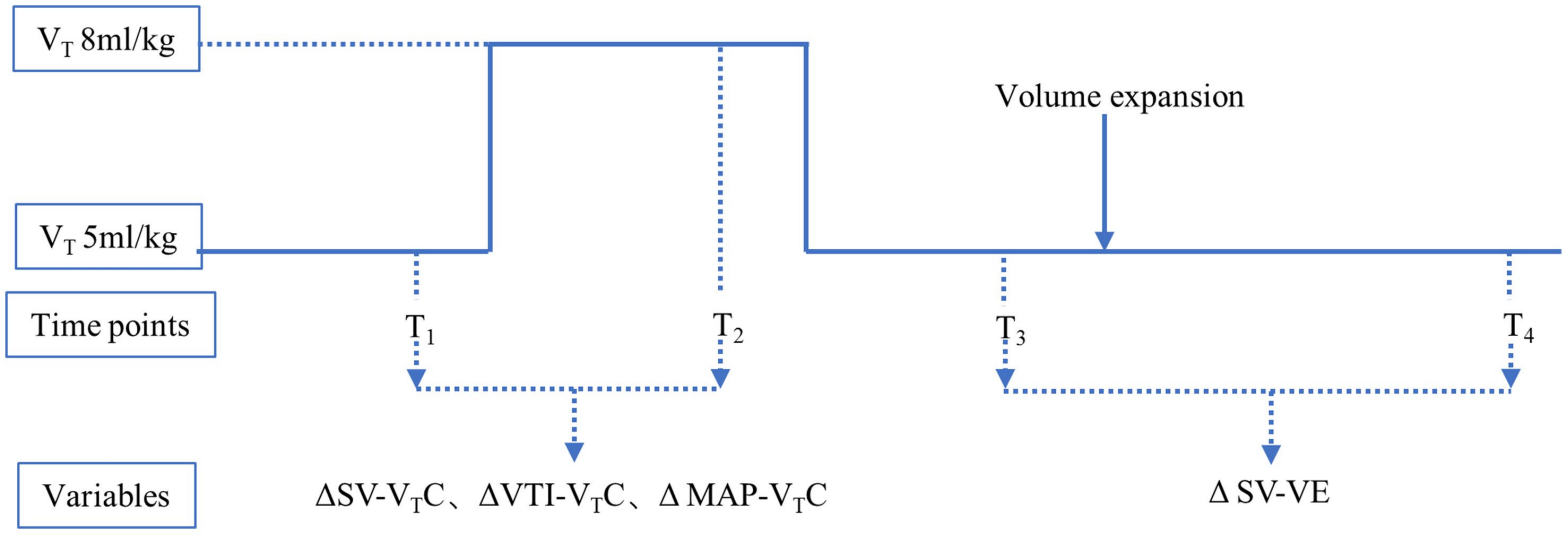
Study protocol. T1, with tidal volume of 5 mL/kg ideal body weight; T2, after application of VTC by increasing the tidal volume to 8 mL/kg ideal body weight for 2 min; T3, after reducingtidal volume back to 5 mL/kg ideal body weight for 2 min; and T4, after volume expansion (by intravenous infusion of 250 mL normal saline over 10–15 min). ΔSV-VTC indicates relative change of SV after VTC; ΔVTI-VTC indicates relative change of VTI after VTC, ΔMAP-VTC indicates relative change of MAP after VTC, ΔSV-VE indicates relative change of SV after volume expansion.

The study protocol (Figure 1) was initiated only after the beginning of the operation (15 min after starting OLV) to minimize the effect of the surgery type on the measured variables. During the study protocol, CO_2_ insufflation was not used in the hemithorax being operated upon. The V_T_C was performed under hemodynamically stable conditions (i.e., heart rate and blood pressure not fluctuating by more than 10% over 1 min before each measurement) without the administration of vasoactive drugs. Transesophageal ultrasound was used to record the inner diameter (D) of the left ventricular outflow tract (LVOT) and velocity time integral (VTI) of the aortic valve in the long axis view of the gastric fundus, in the middle section of esophagus at four time points: T_1_ (with V_T_ 5 mL/kg IBW); T_2_ (after V_T_C application by increasing the tidal volume up to 8 mL/kg IBW for 2 min); T_3_ (after reducing V_T_ back to 5 mL/kg IBW for 2 min); and T_4_ (after volume expansion intravenous infusion of 250 mL normal saline for 10–15 min).

Using the measured indices, the following were calculated:

1) SV was automatically calculated by the ultrasonic instrument
2) ΔSV-V_T_C (change in SV after V_T_C): $\Delta SV-V_{T}C=\left[(SV_{T2}-SV_{T1})÷SV_{T1}\right]\times 100\%$
3) ΔVTI-V_T_C (change in VTI after V_T_C): $\Delta VTI-V_{T}C=\left[(VTI_{T2}-VTI_{T1})÷VTI_{T1}\right]\times 100\%$
4) ΔMAP-V_T_C (change in MAP after V_T_C): $\Delta MAP-V_{T}C=\left[(MAP_{T2}-MAP_{T1})÷MAP_{T1}\right]\times 100\%$
5) ΔSV-VE (change in SV after volume expansion): $\Delta SV-VE=\left[(SV_{T4}-SV_{T3})÷SV_{T3}\right]\times 100\%$

According to the ΔSV-VE, the patients were separated into two groups: responders (ΔSV-VE ≥10%) and nonresponders (ΔSV-VE <10%) (25).

### Statistical analysis

Statistical analysis was performed using SPSS 21.0 (IBM Corp., Armonk, NY, United States). Data were summarized as the means (±standard deviation), medians (interquartile range), or numbers of patients (%). Categorical variables were compared between groups, using the Fisher exact test or the chi-square test. Time-dependent data were compared (between periods in each group and between groups)using the repeated-measures analysis of variance. Preplanned subgroup analysis was conducted using the unpaired *t*-test if the data were normally distributed or the Mann–Whitney *U*-test if they were not normally distributed. All statistical tests were two-sided, and *p* < 0.05 was considered statistically significant. Receiver operating characteristic (ROC) analysis was performed for assessing the abilities of ΔSV-V_T_C, ΔVTI-V_T_C, and ΔMAP-V_T_C to discriminate responders from nonresponders. The best threshold was defined as the point closest to the upper left corner of the ROC curve (26). In addition, the gray-zone approach was applied for ΔSV-V_T_C and ΔVTI-V_T_C. The gray-zone approach is a method used to assess the nonconclusive range of clinical measurements (25, 27). In the first step, bootstrap resampling was performed for ΔSV-V_T_C and ΔVTI-V_T_C, and the “best threshold” was estimated from each bootstrap sample (obtained by ROC analysis); then, the standard deviation of those 1,000 estimated thresholds was used to estimate the standard error (and 95% confidence interval) of the original (on original data set) best threshold estimate. This calculation was performed using MedCalc (MedCalc Software, Mariakerke, Belgium). In the second step, the inconclusive range for each variable was determined to assess the responsiveness, which was calculated as the cutoff value with sensitivity of <90% or specificity of <90%. If the range (95% CI of the best cutoff threshold) calculated from the first step was larger than that calculated from the second step, the values from the first step were selected as the gray-zone values.

### Sample size estimation

The sample size was calculated using PASS (version 15, NCSS, LLC, Kaysville, Utah, United States). Since there are no previous studies related to the application of VTC in patients during OLV, we expect an area under the ROC curve (AUC) for VTC-induced hemodynamic changes to be set at of at least 0.75. This value was compared with the null hypothesis (AUC = 0.50; sample size for negative/positive group = 1). A minimum of 36 patients were needed (type I error of 0.05 and type II error of 0.2).

## Results

### Patient characteristics

**Figure 2:**
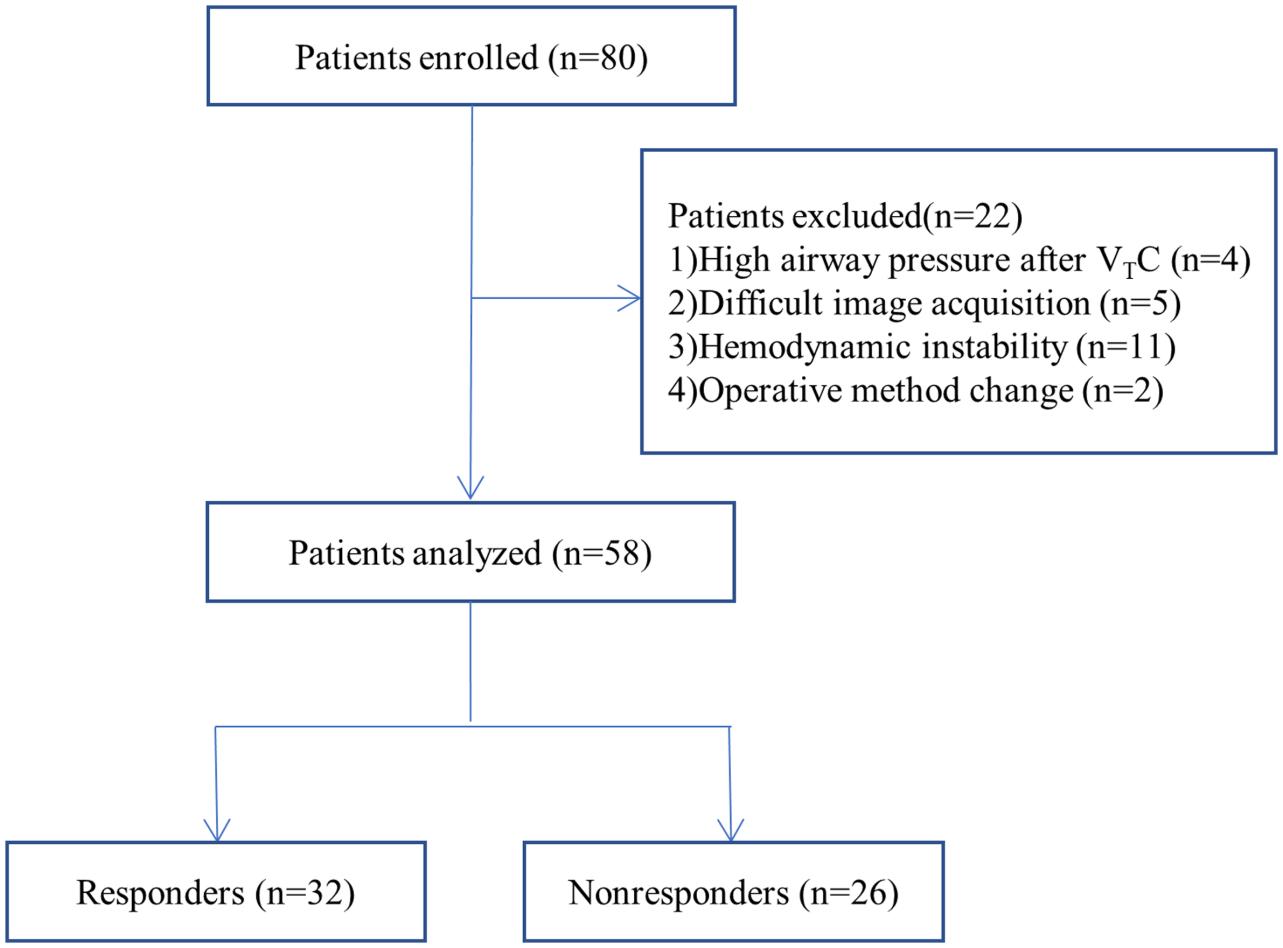
Flow diagram of the study.

Of the 80 patients initially enrolled in the study, 58 completed the study; four patients were excluded because of high airway pressure after V_T_C, five patients were excluded because of difficulty in image acquisition, eleven patients were excluded because of hemodynamic instability during the studyand two were excluded because of operative method change (Figure 2). Table 1 summarizes the characteristics of the patients. Among the 58 patients, 32 (55%) were classified as responders and 26 (45%) as nonresponders. There were no significant differences in characteristics between the two groups (*p* > 0.05).

**Table 1:** Patient characteristics.

	Responders (*n* = 32)	Nonresponders (*n* = 26)	*p-*value
Characteristics			
Age, yr	59 (51–63)	63 (52–67)	0.168
Sex, male/female, *n*	15/17	10/16	0.520
Height, cm	162 ± 6	163 ± 7	0.798
Weight, kg	62 ± 9	64 ± 8	0.291
Ideal body weight, kg	60 (53–69)	57 (55–67)	0.845
ASA physical status I/II/III, *n*	9/20/3	5/16/5	0.477
Driving pressure 5 mL/kg, cmH_2_O	11 (9–13)	12 (11–13)	0.354
Driving pressure 8 mL/kg, cmH_2_O	16 (14–18)	17 (16–18)	0.184
Comorbidities			
Hypertension, *n* (%)	10 (31.3)	10 (38.5)	0.786
Diabetes, *n* (%)	1 (3.1)	5 (19.2)	0.08
Coronary artery disease, *n* (%)	0	2 (7.7)	0.197
Operation			
pneumonectomy/Excision of mediastinal tumor / Excision of lesion of chest wall, *n*	30/1/1	26/0/0	>0.999
Side			
Right side procedure/Left side procedure, *n*	29/3	24/2	>0.999

Data are expressed as mean ± standard deviation ormedian (interquartile range). ASA, American Society of Anesthesiologists.

### Hemodynamic data

**Figure 3:**
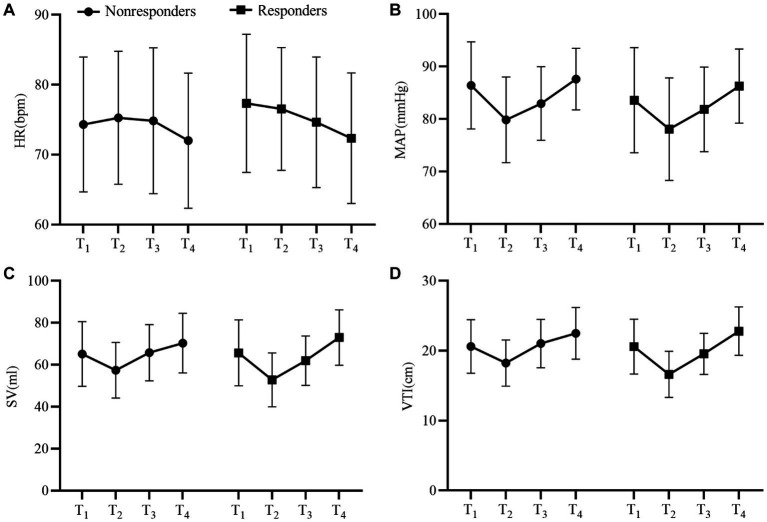
Changes in **(A)** heart rate, **(B)** mean arterial pressure, **(C)** stroke volume, and **(D)** velocity time integral among responders and nonresponders at different time points. T1, with tidal volume of 5 mL/kg ideal body weight; T2, after application of VTC by increasing the tidal volume to 8 mL/kg ideal body weight for 2 min; T3, after reducingtidal volume back to 5 mL/kg ideal body weight for 2 min; and T4, after volume expansion (by intravenous infusion of 250 mL normal saline over 10–15 min).

**Figure 4:**
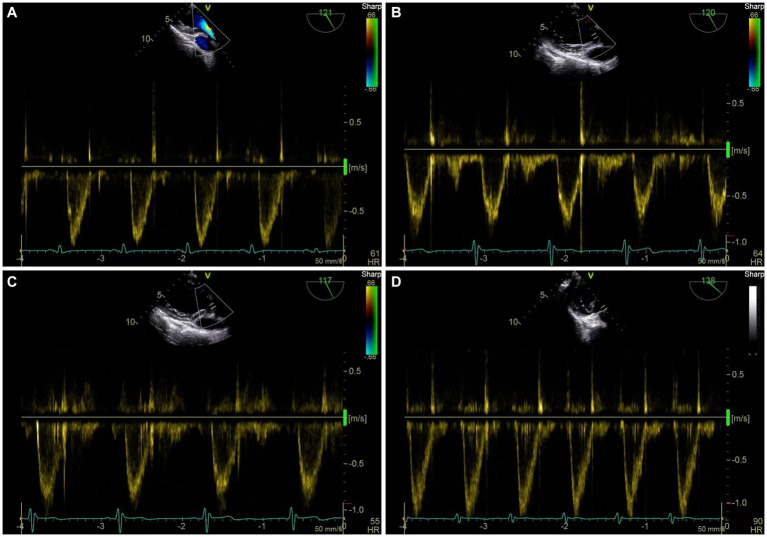
Representative figure of peak systolic velocity at aortic valve before and after VTC and volume expansion as measured by transesophageal Doppler ultrasound. **(A)** Systolic blood flow spectrum of left ventricular outflow tract (LVOT) at aortic valve orifice at T1. **(B)** Systolic blood flow spectrum of LVOT at aortic valve orifice at T2. **(C)** Systolic blood flow spectrum of LVOT at aortic valve orifice at T3. **(D)** systolic blood flow spectrum of LVOT at aortic valve orifice at T4.

Table 2 shows the hemodynamic variables in responders and nonresponders. In both groups, MAP, SV, and VTI decreased significantly after V_T_C and increased significantly after volume expansion. Figure 3 depicts the evolution of SV and VTI in responders and nonresponders during the four-step study period. Figure 4 shows the systolic blood flow spectrum at the aortic valve orifice, as seen through the long axis section of gastric fundus, at different time points. The peak systolic velocity at the aortic valve orifice decreased significantly at T_2_, recovered at T_3_, and then again increased after volume expansion at T_4_.

**Table 2:** Hemodynamic variables at each time point.

	T_1_	T_2_	*p* value T_1_ *vs* T_2_	T_3_	T_4_	*p* value T_3_ *vs* T_4_
HR(beats/min)						
Responders	77 ± 10	77 ± 9	0.387	75 ± 10	72 ± 9	<0.001
Nonresponders	74 ± 10	75 ± 10	0.421	75 ± 9	72 ± 10	0.011
MAP (mmHg)						
Responders	80 (77–90)	77 (72–84)	<0.001	83 (76–88)	88 (80–92)	<0.001
Nonresponders	88 (79–93)	80 (75–84)	<0.001	85 (77–88)	89 (82–92)	<0.001
SV (ml)						
Responders	65 (58–69)	52(47–54)	<0.001	62 ± 12	73 ± 13	<0.001
Nonresponders	61 (56–72)	56 (49–60)	<0.001	66 ± 13	70 ± 14	<0.001
VTI (cm)						
Responders	19.9 (18.3–22.2)	16.0 (14.6–18.0)	<0.001	20 ± 3	23 ± 3	<0.001
Nonresponders	19.1 (17.9–23.0)	16.7 (16.1–19.9)	<0.001	21 ± 3	22 ± 4	<0.001

Data are expressed as mean ± standard deviation or median (interquartile range). T1, with tidal volume of 5 mL/kg ideal body weight; T2, after application of VTC by increasing tidal volume up to 8 mL/kg ideal body weight for 2 min; T3, after reducing tidal volume back to 5 mL/kg ideal body weight and stabilize for 2 min; T4, after volume expansion (with intravenous infusion of 250 mL normal saline over 10–15 min).

### Relationship between hemodynamic changes induced by V_T_C and hemodynamic changes induced by volume expansion

**Figure 5:**
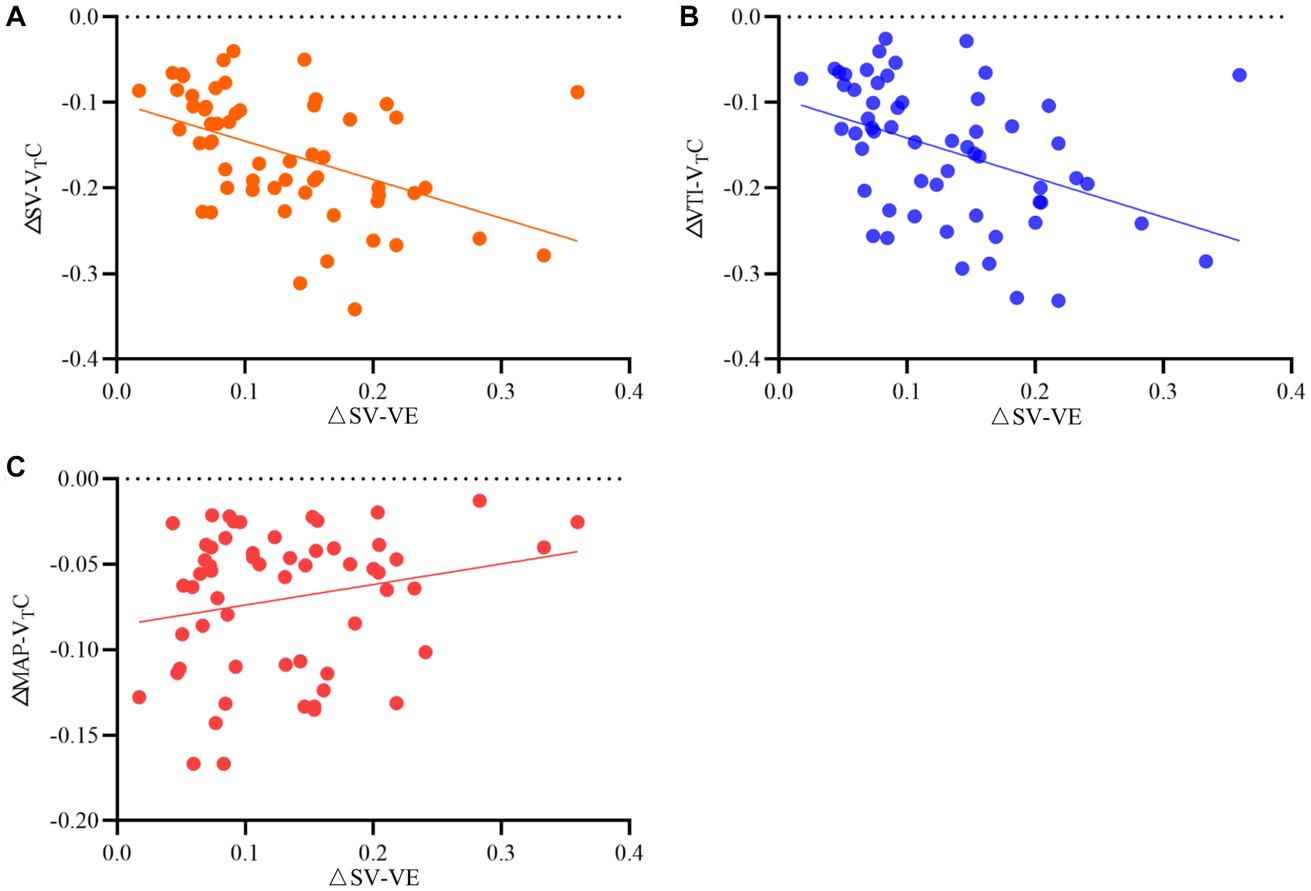
Relationship between relative changes in **(A)** stroke volume (SV), **(B)** velocity time integral (VTI), and **(C)** mean arterial pressure induced by tidal volume challenge (VTC) and by volume expansion (VE). ΔSV-VTC indicates relative change of SV after VTC; ΔVTI-VTC indicates relative change of VTI after VTC, ΔMAP- VTC indicates relative change of MAP after VTC, ΔSV-VE indicates relative change of SV after volume expansion.

Table 3 and Figure 5 show the relationship between ΔSV-V_T_C and ΔSV-VE, ΔVTI-V_T_C, and ΔSV-VE.

**Table 3:** Relationship between relative changes in hemodynamics induced by tidal volume challenge (VTC) and those induced by volume expansion (VE).

Variables	*r*	*R^2^*	*p* value	95% CI
ΔSV-V_T_C *vs* ΔSV-VE	−0.46	0.21	0.0003	−0.64 ~ −0.23
ΔVTI-V_T_C *vs* ΔSV-VE	−0.42	0.18	0.0009	−0.61 ~ −0.18
ΔMAP-V_T_C *vs* ΔSV-VE	0.21	0.04	0.1118	−0.05 ~ −0.45

ΔSV-VTC indicates relative change of SV after VTC, ΔVTI-VTC indicates relative change of VTI after VTC, ΔMAP-VTC indicates relative change of MAP after VTC, and ΔSV-VE indicates relative change of SV after volume expansion.

### Prediction of fluid responsiveness

**Figure 6:**
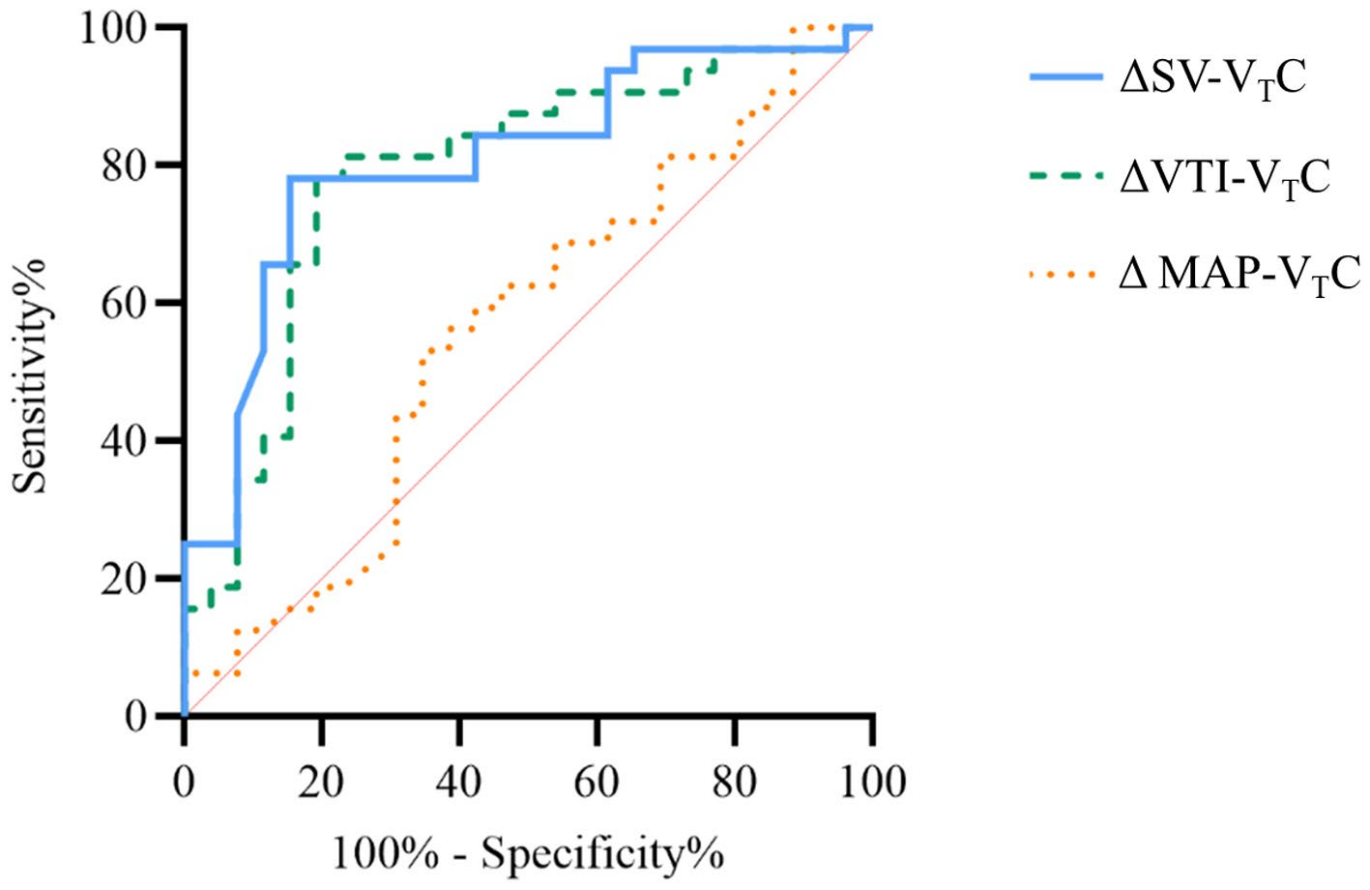
Receiver operating curves generated for changes in stroke volume (SV), velocity time integral (VTI), and mean arterial pressure induced by VTC showing the ability of each to predict the effect of a 250 mL volume expansion given over 10–15 min. ΔSV-VTC indicates relative change of SV after VTC, ΔVTI-VTC indicates relative change of VTI after VTC, and ΔMAP-VTC indicates relative change of MAP after VTC.

Figure 6 shows the predictive values of ΔSV-VTC, ΔVTI-VTC, and ΔMAP-VTC for fluid responsiveness during OLV. A 16.1% decrease in ΔSV-V_T_C predicted fluid responsiveness with sensitivity of 78.1% and specificity of 84.6%; the area under the curve (AUC) for ΔSV-V_T_C to discriminate responders was 0.81 (95% CI, 0.68–0.90). A 14.5% decrease in ΔVTI-V_T_C predicted fluid responsiveness with sensitivity of 78.1% and specificity of 80.8%; the AUC for ΔVTI-V_T_C to discriminate responders was 0.79 (95% CI, 0.66–0.89). A 5.1% decrease in ΔMAP-V_T_C predicted fluid responsiveness with sensitivity of 53.1% and specificity of 65.4%; the AUC for ΔMAP-V_T_C to discriminate responders was 0.56 (95% CI, 0.42–0.69).

### Gray-zone approach for ΔSV-V_T_C and ΔVTI-V_T_C

**Figure 7:**
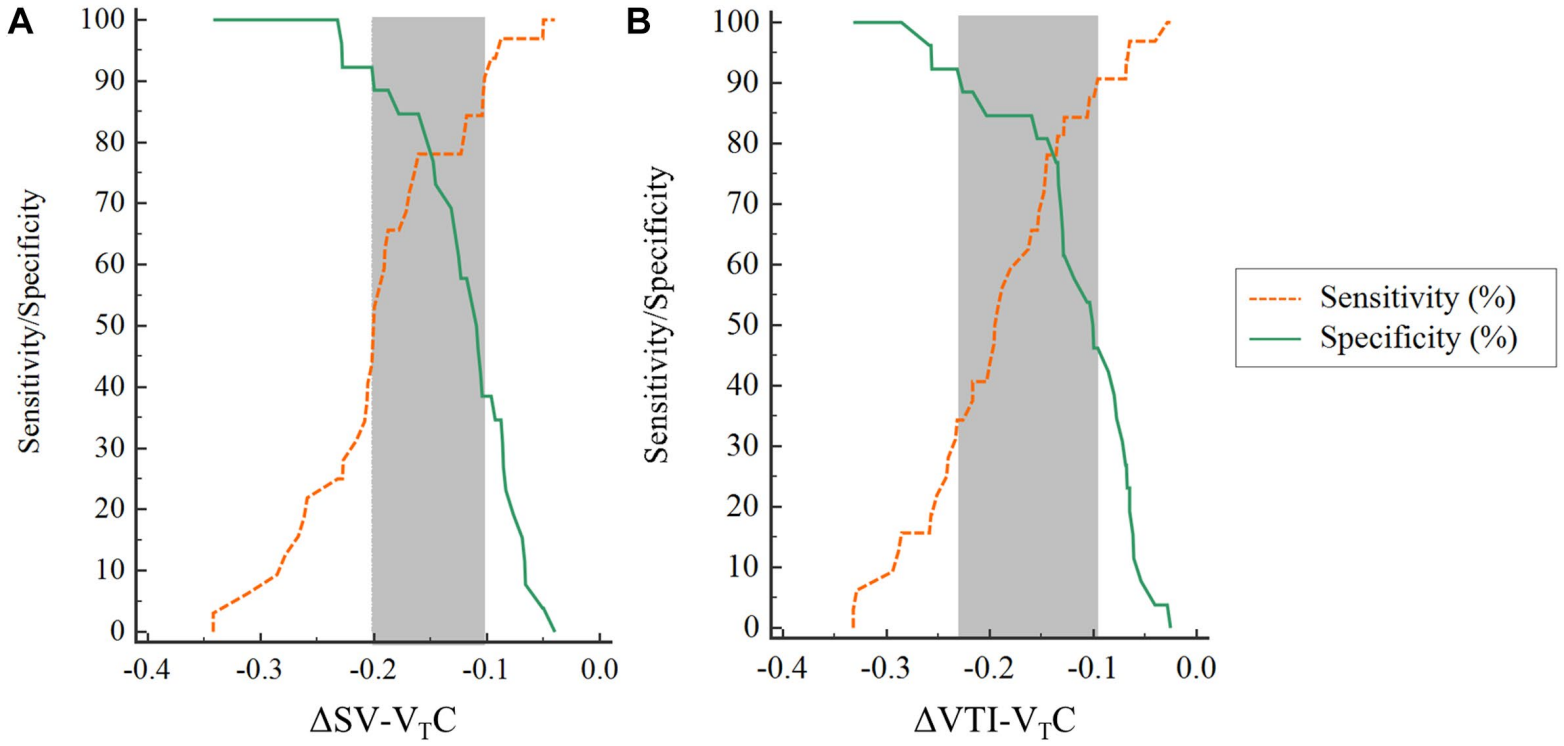
Gray-zone for **(A)** ΔSV-VTC and **(B)** ΔVTI-VTC. The orange and green lines indicate sensitivity and specificity, respectively. The gray zone reveals the inconclusive range for each variable. ΔSV-VTC indicates relative change of SV after VTC, ΔVTI-VTC indicates relative change of VTI after VTC.

The inconclusive region of hemodynamic changes to predict fluid responsiveness was depicted using gray-zone approach. The gray-zone of ΔSV-V_T_C was between −20.1 and −10.21% with 28 numbers of patients (14 responders and 14 nonresponders) (Figure 7A). The gray-zone of ΔVTI-V_T_C was between −22.9 and −9.6% with 29 numbers of patients (17 responders and 12 nonresponders) (Figure 7B).

## Discussion

This study evaluated the value of V_T_C-induced hemodynamic changes for predicting fluid responsiveness in patients receiving OLV and found significant changes in hemodynamics after V_T_C in both responders and nonresponders; in both groups, MAP, SV, and VTI increased significantly after volume expansion. The V_T_C-induced changes in SV and VTI showed significant correlation with the increases in SV after subsequent volume expansion, but the correlation of MAP was poor. ROC curve analysis showed that the V_T_C-induced changes in SV and VTI could predict fluid responsiveness of OLV patients, while the changes in MAP could not.

Increase in intrathoracic pressure leads to decrease in venous return and right ventricular preload. Meanwhile, increase in transpulmonary pressure leads to increase in right ventricular afterload and decrease in right ventricular ejection, which finally results in decreased cardiac output (28). Previous study found thatPEEP induces a decrease in SV, especially in patients with insufficient blood volume (29). This explains why SV, VTI, and MAP decreased to a certain extent when the tidal volume of patients on OLV was increased from 5 mL/kg to 8 mL/kg. When tidal volume, was decreased, the hemodynamics recovered rapidly. These findings suggest that tidal volume loading test could be a reliable functional hemodynamic test to predict fluid responsiveness in patients receiving OLV.

At present, the use of dynamic indicators such as stroke volume variation (SVV) and pulse pressure variation (PPV)for evaluation of fluid responsiveness during OLV is controversial (19, 30). Although there is a mechanism of hypoxic pulmonary vasoconstriction in the process of OLV, part of the blood flow persists in the nonventilated side but is not involved in the respiratory-related changes of SV. Meanwhile, because of pleural cavity opening and other factors, there are no periodic changes related to mechanical ventilation in the nonventilated lung. A previous study reported that when the tidal volume during OLV was changed from 6 mL/kg to 8 mL/kg, the area under the ROC curve improved from 0.648 to 0.776, indicating that the ability of dynamic indicators to predict fluid responsiveness in OLV patients is related to the tidal volume, and that accuracy can be achieved only when the tidal volume is ≥8 mL/kg (20). Lung-protective ventilation is becoming standard intraoperative management for improving postoperative outcomes during OLV (15, 31, 32). In patients receiving lung-protective ventilation, dynamic indices are not very useful. Therefore, a new approach is needed to assess the volume status of patients during OLV.

To overcome the tidal volume–related limitations of dynamic indicators, some researchers proposed the use of V_T_C (tidal volume increase from 6 mL/kg to 8 mL/kg during two-lung ventilation) to evaluate fluid responsiveness in patients receiving low tidal volume ventilation (24, 33). The consensus of Chinese Experts on Perioperative Lung Protection in Thoracic Surgeryrecommends a tidal volume of 4–6 mL/kg for perioperative lung-protective ventilation. Therefore, in this study, the baseline tidal volume was set at 5 mL/kg, and then increased to 8 mL/kg. Volume state decides the hemodynamic response to of V_T_C. V_T_C will increase intrathoracic pressure and the resulting decrease in SV and VTI will make the Frank–Starling curve move to the right; the impact will be more obvious in patients with hypovolemia. However, whether V_T_C can be used to evaluate fluid responsiveness in patients with OLV is unknown. In this study, the tidal volume of OLV patients was increased from 5 mL/kg to 8 mL/kg. The AUCs of V_T_C-induced relative changes of SV and VTI were 0.81 and 0.79, respectively. The sensitivity and specificity were 78.1 and 84.6%, respectively, for SV and 78.1 and 80.8%, respectively, for VTI. A previous study that assessed the usefulness of change in SV for predicting fluid responsiveness during OLV reported an AUC of 0.84 and sensitivity and specificity of 76.5 and 84.6%, respectively (34). A similar study found that the relative change in SVV and PPV induced by PEEP upregulation from 0 cm H_2_O to 10 cm H_2_O can predicted fluid responsiveness in patients during OLV. The AUCs of change in SVV and change in PPV for evaluating fluid responsiveness during OLV were 0.90 and 0.88, respectively; the sensitivity and specificity were 88 and 82%, respectively for change in SVV and 83 and 72%, respectively, for change in PPV (35). These findings suggested that functional hemodynamic tests can accurately predict the volume reactivity of patients with OLV.

Although most studies use ROC analysis to evaluate diagnostic efficiency and determine the cutoff value, sensitivity, and specificity, the method may not accurately reflect the clinical situation (36). ROC analysis can only provide a single cutoff value to separate patients into two types (e.g., responder or nonresponder in this study). However, in our study, “responder” does not necessarily mean that the patient is in a state of hypovolemia. A variety of other factors, such as increased venous volume and decreased venous wall tension, will also lead to the patient being classified as a “responder.” The gray-zone method provides two cutoff values, which is of more practical use (37). This method clearly provides the highest and lowest cutoff values, which allows the clinician to make more rational decisions regarding rehydration in the operating room. When ΔSV-V_T_C or ΔVTI-V_T_C is in the gray-zone between the two cutoff values, there is uncertainty. In our study, 48% of patients were in the gray zone of ΔSV-V_T_C for predicting fluid responsiveness and 50% were in the gray zone of ΔVTI-V_T_C for predicting fluid responsiveness. For patients in the gray zone, a mini fluid challenge can be carried out to observe the changes in hemodynamics. Using gray-zone values instead of a single cutoff value for goal-directed fluid therapy can optimize fluid management and free the clinician from the binary “black–white” decision making of the ROC method.

There are several limitations in the present study. First, the total number of patients may not be sufficient to generalize our result to all patients. Further studies with larger number of patients calculated with precise parameter are needed to describe the physiologic mechanism of V_T_C on dynamic parameters during OLV. Second, the results of this study cannot prove the effectiveness of V_T_C in predicting fluid responsiveness in patients with decreased left ventricular function. Patients with decreased cardiac reserve function are more vulnerable to volume load and need more refined fluid management strategies. In addition, due to the patient’s own condition, ultrasound measurements could not be obtained in few patients in this study, and the use of transesophageal ultrasound may be impractical in many cases due to the limited availability of ultrasound machines or in short-term surgeries.

## Conclusion

In conclusion, the relative changes in SV and VTI induced by V_T_C can accurately predict fluid responsiveness during OLV, but the relative change in MAP induced by V_T_C cannot.

## Data availability statement

The datasets presented in this study can be found in online repositories. The names of the repository/repositories and accession number(s) can be found in the article/supplementary material.

## Ethics statement

The studies involving human participants were reviewed and approved by the Ethical Committee of Northern Jiangsu People’s Hospital, Yangzhou, China. The patients/participants provided their written informed consent to participate in this study.

## Author contributions

YZ and YD: writing original draft. JZ: data analysis. YZ and TH: writing review & editing. JG: supervision and conceptualization. All authors contributed to the article and approved the submitted version.

## Funding

The National Natural Science Fund, China (82172190, 82101299), General Project of Medical Scientific Research Project of Jiangsu Provincial Health Commission (M2021105) and Special Fund for Yangzhou Key Laboratory Cultivation (YZ2021148).

## Conflict of interest

The authors declare that the research was conducted in the absence of any commercial or financial relationships that could be construed as a potential conflict of interest.

## Publisher’s note

All claims expressed in this article are solely those of the authors and do not necessarily represent those of their affiliated organizations, or those of the publisher, the editors and the reviewers. Any product that may be evaluated in this article, or claim that may be made by its manufacturer, is not guaranteed or endorsed by the publisher.
